# Establishment of prognostic models for adenocarcinoma of oesophagogastric junction patients with neoadjuvant chemoradiotherapy: a real-world study

**DOI:** 10.1186/s13014-022-02016-3

**Published:** 2022-03-03

**Authors:** Rongxu Du, Jiao Ming, Jianhao Geng, Xianggao Zhu, Yangzi Zhang, Shuai Li, Zhiyan Liu, Hongzhi Wang, Zhilong Wang, Lei Tang, Xiaotian Zhang, Aiwen Wu, Zhaode Bu, Yan Yan, Zhongwu Li, Yongheng Li, Ziyu Li, Weihu Wang

**Affiliations:** 1 Key Laboratory of Carcinogenesis and Translational Research (Ministry of Education/Beijing), Department of Radiation Oncology, Peking University Cancer Hospital and Institute, Beijing, 100142 People’s Republic of China; 2 Key Laboratory of Carcinogenesis and Translational Research (Ministry of Education/Beijing), Department of Medical Imaging, Peking University Cancer Hospital and Institute, Beijing, 100142 People’s Republic of China; 3 Key Laboratory of Carcinogenesis and Translational Research (Ministry of Education/Beijing), Department of Gastrointestinal Oncology, Peking University Cancer Hospital and Institute, Beijing, 100142 People’s Republic of China; 4 Key Laboratory of Carcinogenesis and Translational Research (Ministry of Education/Beijing), Department of Gastrointestinal Surgery, Peking University Cancer Hospital and Institute, Beijing, 100142 People’s Republic of China; 5 Key Laboratory of Carcinogenesis and Translational Research (Ministry of Education/Beijing), Endoscopy Center, Peking University Cancer Hospital and Institute, Beijing, 100142 People’s Republic of China; 6 Key Laboratory of Carcinogenesis and Translational Research (Ministry of Education/Beijing), Department of Pathology, Peking University Cancer Hospital and Institute, Beijing, 100142 People’s Republic of China

**Keywords:** Adenocarcinoma of the oesophagogastric junction, Neoadjuvant chemoradiotherapy, Pathologic response, Inflammation-based and nutrition-related factors, Prediction models

## Abstract

**Background:**

Multimodal therapies based on surgical resection have been recommended for the treatment of adenocarcinoma of the oesophagogastric junction (AEG). We aimed to evaluate prognostic factors in AEG patients receiving neoadjuvant chemoradiotherapy and to build predictive models.

**Methods:**

T3 − T4N + M0 AEG patients with resectable Siewert type II/III tumours were enrolled in this study. All patients underwent neoadjuvant chemoradiation, followed by radical surgery or systemic therapy according to clinical response. Survival analysis was performed using the Kaplan–Meier method; multivariate analysis using the Cox proportional hazards method was also conducted. The Harrell concordance index (C-index) was used to test the prognostic value of models involving prognostic factors, and consistency between actual and predicted survival rates was evaluated by calibration curves.

**Results:**

From February 2009 to February 2018, 79 patients were treated with neoadjuvant chemoradiotherapy; 60 patients of them underwent radical surgery. The R0 resection rate was 98.3%, and 46.7% of patients achieved a major pathologic response (MPR), namely, a residual tumour issue less than 10%. The 5-year overall survival (OS) rate was 63%, and the 5-year progression-free survival (PFS) rate was 48%. The incidence of grade 3 complications was 21.5%, and no grade 4 complications were reported. According to the results of univariate and multivariate analyses, we included the neutrophil–lymphocyte ratio (NLR), prognostic nutrition index (PNI), eosinophilic granulocyte (EOS) and postoperative pathologic stage in nomogram analysis to establish prediction models for OS and PFS; the C-index of each model was 0.814 and 0.722, respectively. Both the C-index and calibration curves generated to validate consistency between the actual and predicted survival indicated that the models were well calibrated and of good predictive value.

**Conclusions:**

AEG patients achieved favourable downstaging and pathologic response after neoadjuvant chemoradiation, with acceptable adverse effects. Inflammation-based and nutrition-related factors and postoperative pathologic stage had a significant influence on OS and PFS, and the predictive value was verified through prognostic models.

## Background

Oesophagogastric junction (EGJ) carcinoma is defined as carcinoma located 5 cm above and below the oesophagogastric junction. EGJ carcinoma ranks among the most common tumours worldwide and has become the most rapidly increasing tumour in Western countries [1–4]. Adenocarcinoma accounts for a great majority of EGJ carcinomas in East Asia [5–7]. The overall prognosis of this carcinoma is poor; although surgery is considered the fundamental treatment, local control and overall survival (OS) remain unsatisfactory, especially with advanced tumours [1]. Moreover, 70% of EGJ patients develop distant metastasis after primary tumour resection [8].

Given the deficiencies in single treatment, many studies have explored the value of comprehensive therapies for the treatment of AEG. Several prospective randomized control trials [9, 10] have indicated that neoadjuvant radiotherapy/chemoradiotherapy improves local control and survival among patients with potentially curable AEG. Yang et al. [11] compared preoperative and postoperative chemoradiotherapy in advanced gastric cancer, suggesting better OS, progression-free survival (PFS) and treatment compliance for preoperative chemoradiation. However, the application of neoadjuvant radiation or chemoradiation remains controversial. Some studies have suggested that chemoradiotherapy contributes to survival after surgery with limited lymph node dissection [12, 13], though not all patients can achieve optimal tumour regression in clinical practice; thus, selecting patients who may benefit from neoadjuvant chemoradiation is crucial for individualized treatment.

In addition, to provide more sufficient discussion about possible prognostic factors, several inflammation-based and nutrition-related factors, including the neutrophil–lymphocyte ratio (NLR), platelet-lymphocyte ratio (PLR), prognostic nutrition index (PNI), eosinophilic granulocyte (EOS) and fibrinogen (Fbg) [14–16], were explored in our study. Although some of these factors have been reported to have prognostic value for malignant tumours, e.g., a higher EOS value is suggested to be related to better survival in colon cancer [15], their application in AEG has rarely been reported. There are also predictive models involving inflammation-based and nutrition-related prognostic factors in oesophageal and gastric cancers [17, 18], but their conclusions are discordant, and other prognostic factors have rarely been included in these models.

Our study aimed to explore prognostic models, including clinical indices and treatment responses, and to select AEG patients who may benefit based on these models to promote individualized treatment.

## Methods

### Patients

Patients with previously untreated, biopsy-proven, locally advanced AEG were included in this retrospective study. Detailed inclusion criteria were as follows: patients (1) aged 18–75 years old; (2) diagnosis of oesophagogastric junction adenocarcinomas by endoscopy and biopsy pathology; (3) primary tumours of T3–T4 stage and positive lymph nodes; (4) primary tumours considered to be resectable before start of treatment; (5) Siewert II or III type of AEG, according to the definition from Siewert and Stein [19], whereby type II is defined as a tumour invading the EGJ, in which the centre is located between 1 cm above and 2 cm below the EGJ, and type III is defined as a tumour invading the EGJ, in which the centre is located 2 cm-5 cm below the EGJ; (6) Eastern Cooperative Oncology Group (ECOG) score 0 or 1 before start of treatment, with tolerance to chemoradiation; and (7) neutrophil count greater than 1,500 cells/µL, haemoglobin greater than 100 g/L, and platelet count greater than 100,000/µL.

All patients underwent imaging staging and peritoneal washings before the start of treatment. Patients with distant metastases suggested by imaging examination or peritoneal washings were excluded.

### Haematological index measurement

Baseline blood data were obtained by collecting blood from the peripheral vein of each patient within 1 week before neoadjuvant chemoradiation. The EOS and Fbg were obtained directly by the blood test, and the NLR and PLR were defined as the absolute neutrophil count and platelet count divided by the absolute lymphocyte count, respectively. The PNI was calculated using the following formula: 10 × serum albumin (g/dL) + 0.005 × total lymphocyte count (per mm^3^) [20]. The X-tile program was used to determine the optimal cut-off value of the above factors for predicting prognosis [21].

### Assessment

The patients were diagnosed with AEG through endoscopy and biopsy. Endoscopy, endoscopic ultrasonography and computer tomography (CT) were used to determine the location and Siewert type of primary tumours and clinical stage before the start of treatment, for the patients with staging in question, discussion with radiographers would be carried out for more accurate clinical stage. After completion of neoadjuvant chemoradiation, the patients were subjected to clinical response assessment by CT to evaluate the therapeutic effect based on RECIST version 1.1 [22] and determine subsequent treatment. We used the pathological analysis results after surgery as the standard criteria to evaluate the R0 resection rate and pathologic response rate. Patients with less than 10% residual tumour tissue in relation to the macroscopically identifiable tumour bed of the primary tumour site were considered as having a major pathologic response (MPR) [23]. The tumours were staged clinically and pathologically according to the AJCC 8th edition of cTNM stage and ypTNM stage, respectively.

### Treatment

**Fig. 1:**
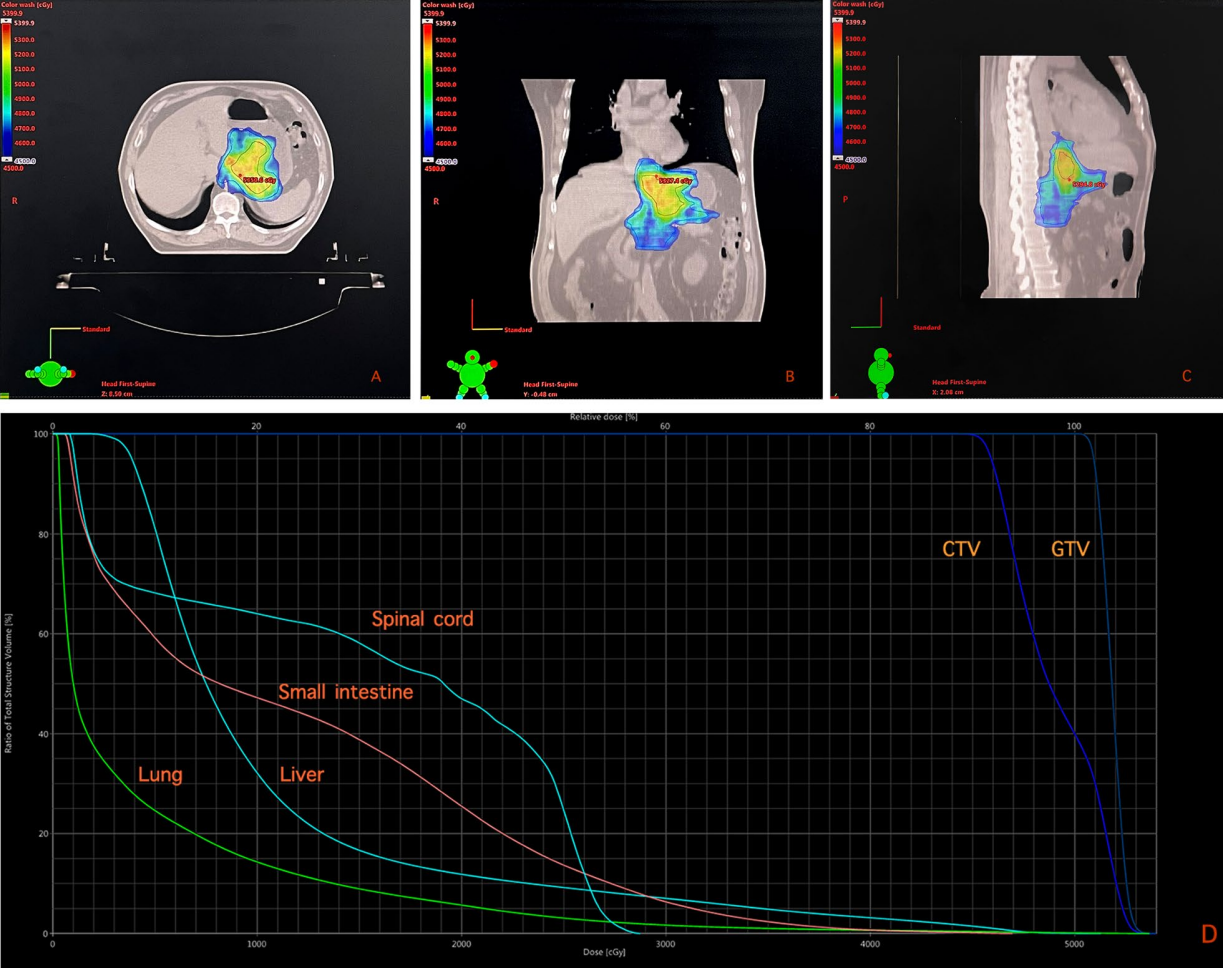
The color wash mapping depicting the radiation dose distribution of a realistic case in the transverse section (**A**), coronal section (**B**), and sagittal section (**C**). The Dose-Volume Histogram (DVH) indicated the dose received by normal tissues (lung, liver, small intestine and spinal cord) and target volumes (**D**)

All the patients gave their written informed consent before any treatments, including radiotherpy, chemotherapy and surgery. All patients enrolled in this study accepted neoadjuvant chemoradiation. Volumetric-modulated Arc Therapy (VMAT) was executed in this study. Cone beam CT (CBCT) was used to verify the planned target volume with actual location of tumors during radiotherapy process, in the first three times of radiation, and once a week from the 2nd week of radiotherapy. The gross tumour volume (GTV) included primary tumour and metastatic lymph nodes; the clinical tumour volume (CTV) included the GTV and high-risk lymphatic drainage area. External beam radiotherapy was applied to the CTV at a total dose of 45 Gy in 25 fractions of 1.8 Gy and the GTV at a total dose of 50 Gy in 25 fractions of 2.0 Gy, 5 times a week. An example of realistic dose distribution and Dose-Volume Histogram was shown in Fig. 1. Patients accepted concurrent chemotherapy during radiation, and the standard regimen in this study was oxaliplatin (intravenous drip, 40 mg/m^2^, d1) combined with S-1 (oral administration, 30 mg/m^2^, bid, d1–d5) weekly since March 2014, before when 17 patients orally took S-1 (25–35 mg/m^2^, bid, d1–d5) weekly.

For those with newly distant metastases suggested by CT evaluation or surgery findings, systemic treatment should be taken into consideration, including systemic chemotherapy with or without local radiotherapy or cytoreductive surgery. If the response evaluation indicates a stable disease (SD), partial response (PR) or complete response (CR) status, according to RECIST v1.1, radical surgery would be taken into consideration, with the standard surgical method being total gastrectomy with D1 + /D2 lymphadenectomy.

### Statistical analysis

Statistical analysis was carried out with SPSS software, version 24.0 (SPSS Inc., Chicago, IL, USA). OS was defined as the time from diagnosis to date of death from any cause or last follow-up, and PFS was calculated from the start of chemoradiation to initial progress, death from any cause or last follow-up without progress. Survival analysis was performed with the Kaplan–Meier method, and the results were compared by the log-rank test. Multivariate analysis of independent risk factors for survival was performed with the Cox proportional hazards method, and logistic regression analysis was conducted for risk factors related to recurrence and metastasis. We used Fisher’s exact test to clarify the relationship between different factors. All variables with significant results by univariate analysis (P < 0.05) were included in the multivariate model. ROC curves were also produced for blood markers to assess their ability to predict prognosis. The Harrell concordance index (C-index) was employed to test the prognostic value of the model involving all the above prognostic factors, and consistency between the actual observed survival rates and predicted survival rates was evaluated by calibration curves.

## Results

### Patients and treatments

From February 2009 to February 2018, we included 79 AEG patients who accepted treatment at Beijing Cancer Hospital. All patients accepted chemoradiation as their first therapy; the median age was 63 years old. The major initial symptoms of these patients were dysphagia and abdominal pain. Seventy-four patients completed the whole radiation course without pause or delay; the other 5 patients failed to complete the radiotherpy continuously due to treatment-related complications. The delay or deficiency of courses was no more than 5 fractions. Ten patients did not accept further treatment after chemoradiation due to personal reasons, and 9 accepted systemic treatment because of distant metastases found in the evaluation after chemoradiation. The details of the patients’ clinical factors are shown in Table 1. From the survival analysis involved the characteristics in this table, we could tell that age, sex, ECOG score, Siewert type, Lauren type, and clinical T/N stage didn't have significant influence on OS or PFS of patients in this study.

**Table 1:** Overview and univariate analysis of the clinical characteristics of the patient cohort

Patient characteristics	N (%) (N = 79)	Log-rank P value (with OS)	Log-rank P value (with PFS)
Age	44–74 years (median age 63 years)	–	–
Sex		0.182	0.229
Male	76(96.2%)		
Female	3(3.8%)		
ECOG score		0.830	0.517
0	66(83.5%)		
1	13(16.5%)		
Siewert type		0.078	0.120
Siewert II	44(55.7%)		
Siewert III	23(29.1%)		
Unavailable	12(15.2%)		
Lauren type		0.652	0.787
Intestinal type	39(49.4%)		
Diffuse type	14(17.7%)		
Mixed type	15(19.0%)		
Undefined	11(13.9%)		
Clinical T stage		0.301	0.999
T3	16(20.3%)		
T4	63(79.7%)		
Clinical N stage		0.450	0.962
N1	19(24.05%)		
N2	41(51.9%)		
N3	19(24.05%)		

ECOG: eastern cooperative oncology group

Ultimately, 60 patients underwent radical surgery: open total gastrectomy (41) and laparoscopic-assisted total gastrectomy (19) with D1 + /D2 lymphadenectomy. Only one of the patients had a positive margin, and the R0 resection rate was 98.3%. Postoperative pathological analysis showed that 8 patients (13.3%) achieved a pathologic complete response (pCR) and that 28 (46.7%) achieved MPR. The number of lymph nodes surgically removed was 23.14 ± 8.67, the number of positive lymph nodes was 0.57 ± 0.83, and 20 patients (33.3%) were diagnosed with local lymph node metastasis. Fifty-five patients (91.7% of patients treated with surgery, 69.6% of all patients) achieved tumour downstaging confirmed by pathological examination. Fifty-nine patients (74.7% of all patients) achieved tumour downstaging confirmed by pathological or imaging examinations. The details of the surgery and surgical results are listed in Table 2. Postoperative pathologic stage was shown to be a significant prognostic factor both in OS (P < 0.001) and PFS (P < 0.001), other variables like surgery method, scope, Tumour Regression Grade (TRG) and postoperative condition of lymph nodes failed to affect the survival of patients significantly.

**Table 2:** Overview and univariate analysis of surgical data and surgery-related outcomes

Surgery-related factors	N (%) (N = 60)	Log-rank P value (with OS)	Log-rank P value (with PFS)
Type of surgical method		0.981	0.562
Open surgery	41(68.3%)		
Laparoscopic surgery	19(31.7%)		
Scope of lymph node dissection		–	–
D1 + lymphadenectomy	3(5.0%)		
D2 lymphadenectomy	57(95.0%)		
Marginal condition		–	–
Negative	59(98.3%)		
Positive	1(1.7%)		
Lymph node metastasis		0.564	0.243
Positive	20(33.3%)		
Negative	40(66.7%)		
TRG grade		0.070	0.100
Grade 0	8(13.3%)		
Grade 1	20(33.3%)		
Grade 2	26(43.4%)		
Grade 3	6(10%)		
Postoperative pathologic stage		0.000	0.000
Stage 0–II	55(91.7%)		
Stage III–IV	5(8.3%)		
Postoperative complications		0.438	0.095
Anastomotic fistula	6(10%)		
Haemorrhage	4(6.7%)		
Infection	7(11.7%)		
No severe complications	43(71.6%)		

TRG: tumour regression grade

### Survival

The median follow-up time was 38.5 months (5–106 months). There were 6 cases of local recurrences and 27 of distant metastases during follow-up. At the end of follow-up, 31 patients had died due to the cancer, 1 patient died due to postoperative complications, and 2 patients died due to other acute diseases. The 5-year OS rate was 63%; the 5-year PFS rate was 48%. The median OS was 85 months, and the median PFS was 49.94 months.

### Complications

Some patients experienced chemoradiation-related toxicity. Seventeen patients (21.5%) had grade 3 toxicities after chemoradiation, but no grade 4 toxicity occurred in this study. Nine patients (11.4%) had grade 3 haematologic toxicities, which ranked as major complication in this study; five patients (6.3%) had grade 3 radiation oesophagitis and one patient (1.3%) had grade 3 radiation pneumonitis; moreover, three patients with grade 2 radiation pneumonitis got postsurgical pulmonary infection or difficulty in weaning, resulting in prolonged hospital stay after surgery. Altogether, seventeen patients (28.3%) experienced postoperative complications needing intervention, and one patient (1.7% of patients who underwent surgery) died of massive haemorrhage in the hospital 2 weeks after surgery.

### Inflammation-based and nutrition-related factors

**Fig. 2:**
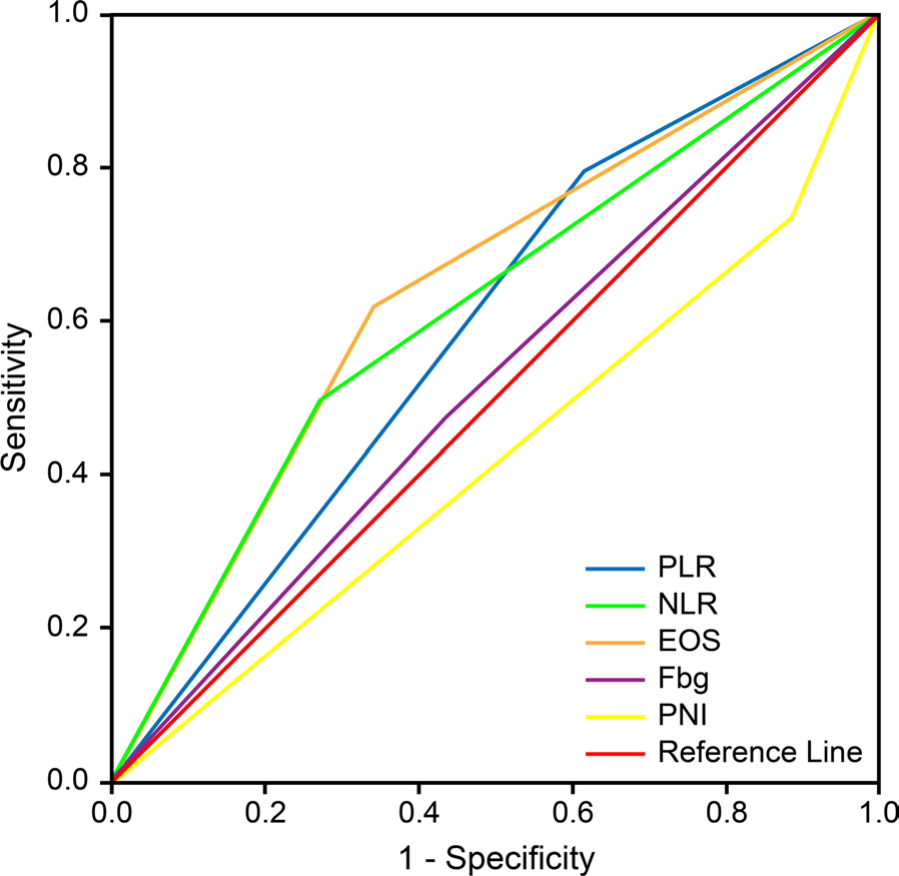
ROC curves of inflammation-based and nutrition-related scores. The area under the ROC curve (AUC) indicated the prognostic value of relevant factors. The EOS (eosinophilic granulocyte) showed an AUC of 0.638 (P = 0.037)

As inflammation-based and nutrition-related factors were considered to be possible prognostic factors in our analysis, we used the X-tile program to determine the cut-off values of NLR, PLR, EOS, PNI and Fbg before we included them in survival analysis, and the results were 2.2, 169.7, 0.1, 55.9 and 345.5, respectively. The cut-off value and corresponding analysis of these factors are listed in Table 3. The cut-off value of EOS was regarded as a best predictive factor, with an area under the ROC curve (AUC) of 0.638 (P = 0.037) (Fig. 2).

**Table 3:** Inflammation-based and nutrition-related factors involved in the analysis

Patient characteristics	N (%) (N = 79)	Log-rank P value (with OS)	Log-rank P value (with PFS)
NLR		0.034	0.048
NLR < 2.2	30(38.0%)		
NLR ≥ 2.2	49(62.0%)		
PLR		0.118	0.149
PLR < 169.7	54(68.4%)		
PLR ≥ 169.7	25(31.6%)		
EOS		0.001	0.070
EOS < 0.1	37(46.8%)		
EOS ≥ 0.1	42(53.2%)		
PNI		0.049	0.078
PNI < 55.9	64(81.0%)		
PNI ≥ 55.9	15(19.0%)		
Fbg		0.540	0.100
Fbg < 345.4	36(45.6%)		
Fbg ≥ 345.4	43(54.4%)		

NLR: neutrophil–lymphocyte ratio; PLR: platelet-lymphocyte ratio; EOS: eosinophilic granulocyte; PNI: prognostic nutrition index; Fbg: fibrinogen

### Analysis of prognosis factors and prediction models

We analysed all related factors possibly involved in patient prognosis. In univariate analysis, NLR (P = 0.034), EOS (P = 0.001), PNI (P = 0.049), and postoperative pathologic stage (P < 0.001) significantly influenced OS; NLR (P = 0.048) and postoperative pathologic stage (P < 0.001) significantly influenced PFS. Multivariate analysis showed that EOS (P = 0.024) and postoperative pathologic stage (P = 0.020) were independent factors related to OS, and postoperative pathologic stage (P < 0.001) significantly influenced PFS.

**Fig. 3:**
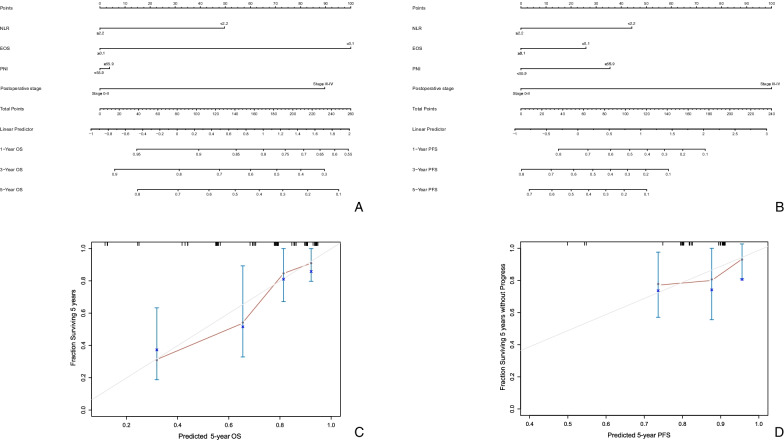
Nomograms predicting OS (**A**) and PFS (**B**) rates of patients in our cohort. The nomogram adds up the points identified on the scale for each independent factor. The total scores projected on the bottom scale indicate the probabilities of 1-year, 3-year and 5-year OS rates and PFS rates. Calibration plots of the nomograms for 5-year OS prediction (**C**) and 5-year PFS prediction (**D**). The X axis displays the nomogram-predicted probability, and the Y axis displays the actual survival rates estimated by the Kaplan–Meier method. The grey line represents excellent calibration, and the red line represents actual calibration. The blue vertical bars indicate 95% CIs

Some inflammation-based, nutrition-related factors as well as postoperative pathologic stage showed a significant influence on OS and PFS in univariate and multivariate analyses, we included as many related factors as possible and integrated them into prognostic nomograms to build prediction models with a high degree of fitness. Each factor was assigned points according to its coefficient. We established prediction models for OS and PFS, and the C-index of each was 0.814 and 0.722, respectively. Calibration curves were generated to validate consistency between the actual survival and the survival probability predicted by the nomograms (Fig. 3). The results indicated that the nomograms were well calibrated.

Since postoperative pathologic stage correlated significantly with both OS and PFS, we further explored the relationship between inflammation-based and nutrition-based factors and downstaging and found that the EOS value was related to postoperative pathologic stage (P = 0.038).

Multivariate analysis also indicated postoperative pathologic stage to be an independent factor related to metastasis (P = 0.004, HR = 0.150, 95% CI 0.041–0.552). In further analysis comparing the effect of postoperative T stage and N stage on metastasis, we found that postoperative T0-T2 stage suggested a much lower risk of metastasis (P = 0.011, HR = 0.119, 95% CI 0.023–0.610). Other factors did not have significant influence on recurrence in situ or distant metastasis in multivariate analysis.

## Discussion

As multimodal therapy has been approved for the treatment of AEG, we evaluated the ability of neoadjuvant chemoradiotherapy to improve the surgical effect and prognosis of patients and screened prognostic factors potentially related to survival. We demonstrated that neoadjuvant chemoradiation contributed to a higher rate of complete resection and potentially improved survival. Additionally, we established prediction models using significant prognostic factors in our analysis.

Routine radical surgery, that is, complete removal of the primary tumour (R0 resection) with lymphadenectomy, has been a mainstay for patients with resectable AEG. Multimodal therapy and complete resection without any residual disease enhance local control and even overall survival, and the quality of surgery is critical for both the short-term and long-term outcomes of patients with AEG [24, 25]. Neoadjuvant therapy has been recommended, especially for patients whose clinical staging suggests a questionable chance of complete tumour removal by primary resection [26]. Total gastrectomy with a transhiatal resection of the distal oesophagus, combined with abdominal D2 lymphadenectomy, is suggested to be the best approach for Siewert II or III tumours [27–29], unless D2 surgery increases surgery-related risk [7].

We explored the effect of neoadjuvant chemoradiation on the results of surgery and prognosis of patients in this study. First, we found a high degree of neoadjuvant treatment completion, 74 of 79 patients completed the scheduled regimen of neoadjuvant therapy consecutively. There was no evidence indicating a remarkable increase in difficulty in the operation after chemoradiation or postoperative complications. There were no grade 4 toxic effects related to neoadjuvant treatment in our study; 78.5% of patients had grade 1 or 2 toxicities, indicating good tolerance to chemoradiation before surgery. The postoperative mortality rate was 1.7%, and the complications morbidity rate was 28.3%, which compare favourably with the good results from western real-world clinical practice [30] and are comparable to the results for patients after radical surgery alone in our centre. Second, pathological analysis showed an ideal effect of neoadjuvant treatment, as R0 resection was achieved in 98.3% of patients, indicating a satisfactory effect of neoadjuvant treatment and high-quality surgery. Since the patients enrolled in our study had advanced primary tumours (T3–T4, mainly T4), suggesting a higher difficulty in achieving a complete response, the MPR rate of 46.7% and pathological downstaging rate of 91.7% still showed a favourable role of neoadjuvant chemoradiation in AEG treatment. A previous phase III trial used neoadjuvant chemotherapy combined with chemoradiation to achieve a pCR of approximately 15% [1]; we achieved a similar effect without long-term induction chemotherapy. Our results also showed that neoadjuvant chemoradiotherapy reduced the lymph node metastasis rate by two-thirds; the number of positive lymph nodes in patients with lymphatic metastasis in our study was also extremely small. Overall, neoadjuvant chemoradiation may help to obtain a higher resection rate and pathologic response, as demonstrated by the postoperative nodal condition and TRG grade.

Overall survival in our study was comparable to that of previous studies: phase II/III trials and real-world studies have reported a 5-year OS rate of 45.0–65.1% [1, 11, 31]. Multivariate analysis showed that postoperative pathologic stage significantly influenced survival. In accordance with some previous studies [32, 33], patients who remained at stage III-IV without downstaging after neoadjuvant treatment had poorer survival. The reason may be that the pathological status of primary tumours and metastatic lymph nodes represents a response to neoadjuvant treatment; patients with a poorer therapeutic response and poorer tumour necrosis might be resistant to the treatment, with a tendency to maintain a higher stage and more residual tumour than patients with a good response. It has been argued that the overall survival of patients whose disease does not respond to neoadjuvant therapy might even be worse than that of patients who accepted surgery alone because adequate surgery is substantially delayed [34, 35].

We generated prediction models for OS and PFS using nomograms and obtained a high C-index for each model, indicating high predictive value. Calibration curves confirmed consistency between the predicted and actual trends, and the results showed that the nomograms were well calibrated. Overall, inflammation-based and nutrition-related factors and postoperative pathologic stage appear to contribute to establishing an applicable prediction model.

Among all factors included in the nomograms, we found some haematological indices to be consequential in predicting the prognosis of AEG carcinoma. Since the inflammation caused by tumours can lead to DNA damage, uncontrolled cell growth and micrometastasis, inflammation markers may be related to survival [36]. NLR and PLR are common inflammation-based factors for various digestive carcinomas [18], but the value of NLR and PLR in predicting prognosis was not unified in previous studies. NLR showed a significant influence in univariate analysis in this study and was included in the nomogram analysis. Yu et al. [16] recognized that serum fibrinogen correlates positively with advanced tumour stage and poor survival in gastric cancer and that the preoperative serum fibrinogen level might be an independent risk factor; however, this factor failed to show value in predicting AEG prognosis in our study. Preoperative nutritional status has been reported to be one of the critical factors for patient outcomes in a variety of surgeries, especially in gastrectomy. PNI is an important index evaluating both immune and nutrition status. Han et al. [37] suggested that AEG patients with a high PNI have a longer OS. The mechanism might be explained in two ways: first, a high level of PNI might indicated a good nutritional condition, resulting in better tolerance to treatment and better outcomes [17]; second, the lymphocyte count is part of the PNI score, and a low lymphocyte level might be associated with immunosuppression, which can lead to tumour progression or recurrence of residual tumours [38]. PNI functioned as a predictive factor in our models and should be evaluated in future studies. Notably, EOS is reported to be a possible predictor for the prognosis of colorectal cancers, whereby it is higher in colorectal carcinoma patients with better prognosis [15]. Studies on the value of EOS in other types of carcinomas are limited. Our study provides more evidence on the effect of EOS on patient survival, as our results showed that a higher level of EOS indicated better overall survival; it was also significantly related to postoperative stage. In summary, NLR ≥ 2.2, EOS ≥ 0.1, PNI < 55.9 and postoperative pathologic stage 0-II might indicate positive OS and PFS outcomes. The prognostic factors above may all be regarded as valuable in prediction models, and how to apply them in screening patients with potential benefits needs to be confirmed in future studies.

There were some limitations in our study. The heterogeneity of retrospective studies is considered a major factor, as selection bias and recall bias are difficult to eliminate, and a prospective study is needed to verify our models. Additionally, our sample size limited the precision of analysis. Expanding the sample size and long-term follow-up are needed to obtain more specific outcomes and screen patients with potential benefits.

## Conclusions

Our study showed a favourable downstaging and pathologic response after neoadjuvant chemoradiation in AEG patients, with acceptable adverse effects. Inflammation-based and nutrition-related factors, such as NLR, EOS, PNI, and postoperative pathologic stage, contribute to establishing an applicable prediction model of survival.

## Data Availability

All data generated or analyzed during this study are included in this published article and its supplementary information files.

## Abbreviations

AEG: Adenocarcinoma of the oesophagogastric junction
MPR: Major pathologic response
OS: Overall survival
PFS: Progression-free survival
NLR: Neutrophil–lymphocyte ratio
PNI: Prognostic nutrition index
EOS: Eosinophilic granulocyte
EGJ: Oesophagogastric junction
Fbg: Fibrinogen
ECOG: Eastern Cooperative Oncology Group
CT: Computer tomography
VMAT: Volumetric-modulated Arc Therapy
CBCT: Cone beam computer tomography
GTV: Gross tumour volume
CTV: Clinical tumour volume
SD: Stable disease
PR: Partial response
CR: Complete response
TRG: Tumour regression grade
AUC: Area under the ROC curve
